# The Influence of the COVID-19 Pandemic on the Prevalence, Characteristics, Management, and Outcomes of Acute Appendicitis at the Academic Tertiary Center, Jeddah

**DOI:** 10.7759/cureus.31968

**Published:** 2022-11-28

**Authors:** Bsaim A Altirkistani, Waleed T Batais, Renad M Alsolamy, Bandar Y Sulaimani, Ghada O Bakhidar

**Affiliations:** 1 College of Medicine, King Saud Bin Abdulaziz University for Health Sciences, Jeddah, SAU; 2 College of Medicine, King Abdullah International Medical Research Center, Jeddah, SAU; 3 Department of Emergency Medicine, King Abdulaziz Medical City, Jeddah, SAU

**Keywords:** pandemic, covid-19, appendectomy, abdominal pain, acute appendicitis

## Abstract

Background: Acute appendicitis is the leading cause of acute abdominal pain that requires immediate intervention. Nonetheless, during COVID-19, hospital visits decreased as a result of serious COVID-19 concerns at that time, resulting in a decreased number of diagnosed cases with acute appendicitis due to COVID-19 restriction issues.

Objectives: To report the percentage numbers, characteristics, applied management, and outcomes of patients with acute appendicitis during the COVID-19 pandemic and compare them to pre-COVID-19 cases.

Methods: A retrospective cohort study included all patients with acute appendicitis in the determined periods “pre-COVID-19” and “during COVID-19“ at King Abdul-Aziz Medical City, Academic Tertiary Center, Jeddah, Saudi Arabia. Mean and standard deviation were used, while categorical data were reported as frequencies and percentages. Variables were analyzed by the Chi-squared test, Fisher’s exact test, and Mann-Whitney test as appropriate.

Results: A total of 298 patients were included. The period of the pre-COVID-19 pandemic had 161 (54%) patients, while 137 (46%) were identified during COVID-19. The number of laparoscopic appendectomies performed during COVID-19 was less than the pre-COVID-19 pandemic of 96 cases (70.1%) vs 133 cases (82.6%) (P=0.0106). Uncomplicated appendicitis was the most commonly reported type of appendicitis in both periods: 113 (82.5%) during COVID-19 vs 135 (83.9%) pre-COVID-19, (P=0.7526). Furthermore, the number of patients who presented to the ER between 24 and 48 hours after the onset of symptoms was similar before and during the pandemic: 111 (68.9%) vs 89 (65%).

Conclusion: Overall, we conclude that during the COVID-19 period, there was a reduction in the number of patients presenting with acute appendicitis and a lower chance of undergoing laparoscopic appendectomy due to COVID-19 restrictions. There was also an increase in perforated appendicitis and a decrease in gangrenous appendicitis.

## Introduction

Acute appendicitis is the most common cause of acute abdominal pain that requires surgical intervention [1]. It is defined as an inflammation of the vermiform appendix often due to luminal obstruction leading potentially to increased wall tension and perforation. Patients with acute appendicitis often present with the migration of periumbilical pain to the right lower quadrant [2]. In the 21st century, the pooled incidence of acute appendicitis cases was 100 per 100,000 person-years in Northern America, with 378,614 cases reported in 2015. While in Western and Eastern Europe, the pooled incidence was 151 and 105 per 100,000 person-years, respectively [3].

On March 11, 2020, COVID-19 has been globally declared a pandemic health issue by the World Health Organization (WHO) [4]. It had resulted in serious concern due to its vague characteristics and methods of transmission at that time. Fair of acquiring infections and the applied COVID-19 restrictions all may be contributed to the decrease in the number of hospital visits during the pandemic. Additionally, healthcare facilities were encouraged and recommended to postpone elective surgeries, especially for those with less severe complaints, to minimize the exposure risk for such patients as much as possible [5,6]. Thus, making a decision in such circumstances is challenging and debatable for doctors as to whether to go for surgery or conservative options during the pandemic [7]. A study conducted in December 2019 and April 2020 reported that a 40.7% decrease was noted in the weekly incidence of acute appendicitis [6]. Also, a slight 5% decrease in the incidence of acute appendicitis during the pandemic was observed in 21 hospitals. Moreover, a higher percentage of complicated appendicitis was reported, and patients were more likely to present after more than 24 hours since the symptoms’ onset during the pandemic [5,8]. Therefore, the rapid changes in practice that occurred due to COVID-19 resulted in the need for more investigations into how it has affected certain surgical management plans and outcomes, including the care of acute appendicitis.

Nevertheless, after almost two years since the start of the first case of COVID-19 in the world, there is a lack of studies discussing the effects of the COVID-19 pandemic on acute appendicitis management and outcomes, especially in Saudi Arabia. Therefore, the aim of this study is to estimate the prevalence, characteristics, applied management, and outcomes of patients with acute appendicitis during COVID-19 in comparison to pre-COVID-19 cases in an academic tertiary center.

## Materials and methods

Study settings and design

This is a retrospective study design aimed to target patients with acute appendicitis presented to King Abdul-Aziz Medical City, Academic Tertiary Center, Jeddah, Saudi Arabia.

Identifications of study participants

Patients with acute appendicitis between July 2018 and October 2021 were included. The first case of COVID-19 spotted in Saudi Arabia was in March 2019, and thereafter, it has been declared a worldwide pandemic. While October 2021 was the month when all COVID-19 restrictions were first lifted in Saudi Arabia. Therefore, patients with acute appendicitis were classified into two groups based on whether the pandemic has started or not in Saudi Arabia. So, those with acute appendicitis presenting to the emergency department between July 2018 and February 2020 were classified into a pre-COVID-19 pandemic (group A), while those presenting between March 2020 and October 2021 were under the COVID-19 pandemic (group B). Patients were identified using the International Classification of Diseases (ICD-10) codes. 

Data collection process

The data were obtained from the electronic medical records of the academic tertiary center using the Best Care 2.0 System (ezCaretech, BESTCare System, Seoul, South Korea). A data collection form was designed and contained the study variables, such as demographic and clinical data. For example, age, gender, period of presentation, and laboratory values were collected. Additionally, the length of symptoms prior to emergency presentation, treatment modalities, and grade of appendicitis were also obtained. Afterward, the collected data were entered into a Microsoft Excel (Microsoft Corporation, Redmond, WA, USA) file by the research team and kept confidential. 

Statistical analysis

Continuous variables were reported using the mean and standard deviation. While categorical variables were reported as frequencies and percentages. Variables were analyzed by the Chi-square test, Fisher’s exact test, and T-test as appropriate. Statistical significance was defined as P<0.05. Statistical analysis was performed using JMP Statistical Software (SAS Institute, Cary, NC, USA) a subsidiary of SAS Institute, version 15.2.0.

## Results

Demographics and clinical characteristics

A total of 298 patients were identified and included between the two specified periods. In group A, there were a total of 161 patients (mean age, 26.07; SD, 13.59), of which 104 (64.6%) were male, and a total of 137 patients (mean age, 26.85; SD, 15.63) were included in group B, of which 80 (58.4%) were male (P=0.2723). Moreover, the number of patients who presented to the ER between 24 and 48 hours after symptom onset in group A was 111 (68.9%) compared to 89 (65%) in group B (Table 1). 

**Table 1:** A comparison between pre-COVID-19 and during COVID-19 on demographics and clinical characteristics.

	Pre-COVID 161 (54%)	During COVID 137 (46%)	P-value
Age, mean (SD)	26.07 (13.59)	26.85 (15.63)	0.9532
Gender, female	57 (35.4%)	57 (41.6%)	0.2723
Gender, male	104 (64.6%)	80 (58.4%)	
Length of symptoms, <24 h	15 (9.4%)	19 (13.9%)	0.1441
Length of symptoms, 24-48 h	111 (68.9%)	89 (65%)	
Length of symptoms, 48-72 h	13 (8.1%)	18 (13.1%)	
Length of symptoms, >72 h	22 (13.6%)	11 (8%)	

Management and outcomes

For the management of appendicitis, laparoscopic appendectomy was performed in 133 (82.6%) cases in group A, while it was performed in 96 (70.1%) patients in group B, with a statistically significant difference (P=0.0106). In addition, 19 (11.8%) patients had an open appendectomy in group A in contrast to group B with 27 (19.7%) patients, (P=0.0597). Furthermore, the number of patients who re-visited the ER after hospital discharge for a similar presentation was 14 (8.7%) in group A vs 10 (7.3%) in group B (P=0.6769). Only four (2.5%) patients of group A required re-admission vs six (4.4%) patients of group B, (P=0.5213), Table 2. 

**Table 2:** A comparison between pre-COVID-19 and during COVID-19 on management and outcomes. *P<0.05.

N (%)	Pre-COVID 161 (54%)	During COVID 137 (46%)	P-value
Laparoscopic appendectomy	133 (82.6%)	96 (70.1%)	0.0106*
Open appendectomy	19 (11.8%)	27 (19.7%)	0.0597
Conservative management	9 (5.6%)	15 (11%)	0.1335
Uncomplicated appendicitis	135 (83.9%)	113 (82.5%)	0.7526
Perforated appendicitis	8 (5%)	15 (11%)	0.0797
Gangrenous appendicitis	10 (6.2%)	2 (1.5%)	0.0420*
Abscess	4 (2.5%)	5 (3.6%)	0.7370
Re-visited ER for similar concerns after hospital discharge	14 (8.7%)	10 (7.3%)	0.6769
Re-admission	4 (2.5%)	6 (4.4%)	0.5213

Length of symptom and grade of appendicitis

For those who had uncomplicated appendicitis, 135 (83.9%) patients were in group A vs 113 (82.5%) in group B, (P=0.7526). Whereas, for perforated appendicitis, eight (5%) cases were found in group A vs 15 (11%) group B cases, (P=0.0797). Also, gangrenous appendicitis in group A was found among 10 (6.2%) patients vs two (1.5%) in group B, with a statistically significant difference (P=0.0420), as shown in Table 2. Moreover, during COVID-19, six (40%) patients with perforated appendicitis had presented to the ER within 48-72 hours of symptoms onset, while only three (20%) presented within 24-48 hours with a statistically significant difference, (P<0.0001). On the contrary, among uncomplicated appendicitis patients, 79 (69.9%) of them presented to ER within 24-48 hours of symptom onset (P=0.0001), Table 3. 

**Table 3:** Comparison of length of symptom and grade of appendicitis. *P<0.05.

Length of symptoms	Pre-COVID-19		During COVID-19	
	N (%)	P-value	N (%)	P-value
Uncomplicated appendicitis				
<24 h	14 (10.4%)	0.0100*	18 (15.9%)	0.0001*
24-48 h	98 (72.6%)		79 (69.9%)	
48-72 h	8 (5.9%)		12 (10.6%)	
>72 h	15 (11.1%)		4 (3.6%)	
Perforated appendicitis				
<24 h	-	0.8407	1 (6.7%)	<0.0001*
24-48 h	6 (75%)		3 (20%)	
48-72 h	1 (12.5%)		6 (40%)	
>72 h	1 (12.5%)		5 (33.3%)	
Gangrenous appendicitis				
<24 h	-	<0.0003*	-	0.3981
24-48 h	2 (20%)		1 (50%)	
48-72 h	4 (40%)		1 (50%)	
>72 h	4 (40%)		-	
Abscess				
<24 h	-	0.0284	-	0.0095*
24-48 h	1 (25%)		2 (40%)	
48-72 h	-		-	
>72 h	3 (75%)		3 (60%)	

## Discussion

During the COVID-19 pandemic, many aspects of clinical practice have significantly changed. Investigating such changes might be beneficial in further understanding the disease outcomes and evaluating the current management practices and plans. This study aimed to investigate the prevalence, characteristics, applied management, and outcomes of patients with acute appendicitis during COVID-19 in comparison to the pre-COVID-19 period.

Number of acute appendicitis

Compared to the pre-COVID-19 period, our findings demonstrated a reduction in the number of patients presenting with acute appendicitis during the COVID-19 period, from 161 to 137 patients. Such observation was also noted by several other investigators, Scheijmans et al., who reported a reduction in the rate of acute appendicitis among their patients during the pandemic [8-10]. This decrease can possibly be attributed to patients not seeking medical attention because of the fear of acquiring COVID-19 in hospitals. Tankel et al. also hypothesized that national policies of curfew and self-isolation might have impacted people’s ability to access healthcare services. However, since the decrease noted in this study can be described as a slight decrease, normal causes, such as seasonal variation, can also be a possible explanation for the drop [6].

Length of symptoms

Due to the limited access to healthcare and reduced number of acute appendicitis presentations, it would be assumed that delayed presentation to the ER would be more common during the COVID-19 period. However, our results showed that there was no statistical difference between the two periods in the length of symptoms prior to presentation, with most patients presenting between 24 and 48 hours of symptom onset in both periods. Bosak Veršić et al. noted that most patients before and during the pandemic presented within the first day [11]. Our findings, however, contradict what was stated by El Nakeeb et al., who reported higher rates of delayed presentation during the pandemic [10]. This difference can be explained by the differences in sample size and characteristics since El Nakeeb et al. included a larger sample size from multiple centers [10].

Grades of acute appendicitis

Our study results revealed increasing rates of reported perforated appendicitis cases in group B, and this could be a result of the patient's behaviors during the pandemic. Patients’ attitudes toward seeking medical help during COVID-19 have changed, as most of them defer medical consultation if possible. In fact, most of the perforated appendicitis in group B presented to the ER within 48-72 hours and >72 hours of symptoms onset vs those in group A who presented within 24-48 hours. This delay in seeking medical help has been observed by other investigators too, including Somers, El Nakeeb, and Scheijmans et al., and it is most likely to be associated with a more complicated clinical course [8-10]. 

Management of acute appendicitis

In regard to management, this study has found a statistically significant difference between both periods. A higher chance of undergoing laparoscopic appendectomy was noted among group A patients than those in group B. Similarly, a meta-analysis reported that the rate of open appendectomies during the pandemic was higher in comparison to laparoscopic procedures [12]. Since laparoscopic appendectomies are known to have fewer complications, a shorter length of stay and a faster recovery, such a rise in open appendectomies seems unusual, according to Snyder et al [13]. This rise can be explained by laparoscopic appendectomies being deemed an aerosol-generating procedure early in the pandemic, causing more surgeons to perform open appendectomies, according to Köhler and Di Saverio et al [12,14-15].

ER re-visit and re-admission

This study found no significant differences in the number of patients re-visiting the ER with similar symptoms after discharge or in the rates of re-admission between the two periods. Re-admission rate was also noted to be similar by other studies compared to the two periods [16,17]. El Nakeeb et al., however, reported higher rates of re-admission during the pandemic period. This can be attributed to having a higher rate of complicated cases in their included patients [10].

Limitations and recommendations

It is a single-center study that limits the generalizability of the results in regard to the influence of the COVID-19 pandemic on acute appendicitis. Therefore, a larger scale study would be beneficial in which it may involve all centers of Saudi Arabia. Also, addressing patients' and the public's concerns are vitally important as well as educating them regarding the risk of not seeking timely appropriate care for such a health condition due to the raised concerns from pandemics.

## Conclusions

Finally, during the COVID-19 period, there was a reduction in the number of patients presenting with acute appendicitis, with a lower chance of undergoing laparoscopic appendectomy. There was an increase in perforated appendicitis and a decrease in gangrenous appendicitis.

Therefore, having current data regarding the trends of acute appendicitis is highly crucial, as it gives insight into the effect of such a pandemic and provides lessons to be learned in preparation for future pandemics. This study mainly presents a Saudi perspective on changing trends in acute appendicitis that would help Middle Eastern and international researchers in exploring the effect of the COVID-19 pandemic on such important emergency surgical cases.
